# Supplementary material for: Relationship between C-reactive protein-albumin ratio and prognosis in pediatric patients with severe burns
Source: PLoS One. 2025 May 6;20(5):e0321215. doi: 10.1371/journal.pone.0321215 (PMC12054902; doi:10.1371/journal.pone.0321215)
Supplement: Attachment — Submitted filename: Response to Reviewers.docx [file pone.0321215.s002.docx]

**Response to Reviewers**

**Reviewer 1:** In this single-center retrospective cohort study of 150 pediatric burn patients with severe oil burns, the authors evaluated the relationship between hospitalization duration and albumin, CRP, and CRP-albumin ratio values at time of admission. The results suggested that lower albumin levels, higher CRP levels, and higher CRP-albumin ratios at time of admission were associated with longer hospitalizations. The authors’ aim is an interesting question and one that could be readily applied at the bedside given that these are commonly obtained values. However, this manuscript has several major weaknesses. It requires significant copyediting and has multiple grammatical errors. The only outcome measure that was analyzed in this study was duration of hospitalization, which was used to extrapolate predictions about “prognosis.” Fortunately, there were few deaths in their database, but these patients were excluded which further limits the study and its ability to evaluate eventual outcomes given that mortality was excluded. The study only included oil burns, while excluding thermal and electrical burns or patients with inhalational injuries, which limits generalizability. Additionally, as mentioned in the discussion, a limitation was that burn area localization was not accounted for; I would add that there was no mention of total body surface area which is an important factor in severity of burns. Overall, the conclusions drawn in lines 98-100 stating that the study showed that clinical prognosis in the pediatric burn population can be predicted by the CRP-albumin ratio feels overstated.
The abstract overall does a good job summarizing the key points of the article. The results section here does feel slightly redundant.

Overall, I think this introduction would be a lot stronger if the interesting pathophysiology explanations in the discussion section were moved to the introduction. See my comment #12 below.
Line 24: consider rewording this sentence without the use of the word “important”

Line 29: consider being more specific about timeframe of CRP increase instead of “increases in a short time”

Lines 32-33: utilization of word “important” twice in this sentence.

Lines 39 and 48: instead of “third-degree and above severe burns,” consider rephrasing to “third- and fourth-degree burns”

Line 47: I am a little confused about the inclusion of patients who were treated at your medical center’s burn clinic. Were they all admitted from clinic?

Lines 54-55: I would be interested to hear more about the data regarding patients who died and why it was excluded. What was the number of patients?

Lines 56-57: Why were these groups excluded? Curious about why the focus on oil burns only and why inhalation burns were excluded.

Line 76-77: This could be moved to the “Patients and data” section.

Lines 78-89: In the results section, I would have liked to have seen the numerical lab values included in parentheses for the second and third paragraphs. These read more like a discussion section.

Lines 101-107, 115-117, 125-131:

This is all very interesting and well-researched background that I think belong in the introduction section.

Lines 139-142: This sentence is copied word-for-word directly from the results section.

Lines 146-149: This sentence is difficult to follow initially because “these patients” are referenced before the group (pediatric burn patients who died) is specified.

**Response to reviewer 1:** Editing was done for grammatical errors. In the material method section (Patients with thermal and electrical burns were included in the study. Patients with inhalation burns were not included in the study group), it was stated that thermal and electrical burns were among the inclusion criteria in the study.

Lines 98-100 were revised in line with reviewer suggestions. Abstract conclusion section was revised.

The word "important" in Line 24 was removed.

Line 29 was revised.

The words "important" in Line 32-33 were removed.

Lines 39-48 were revised.

Line 47 was revised.

Lines 54-55: ‘’Three of the burn patients admitted during the time period in which the study was examined (one burn caused by a house and two by a lightning strike, respectively) died in the first 24 hours. Three patients died after the first 24 hours (one burn caused by a flame and two burns caused by hot water, respectively). Since the number of patients who died in the burn center after 24 hours was only three and was not considered sufficient for statistical evaluation, the effect on mortality could not be examined’’ was added to the limitation section.

Lines 56-57: One of the patients who died within the first 24 hours was excluded from the study because of accompanying inhalation burns.

Lines76-77: Since this is a finding of this study, we have indicated it in the ‘’Results’’section. However, we can move it if the editor deems it appropriate.

Lines 78-89 were revised.

Lines 101-107, 115-117 were removed to introduction.

Lines 139-142 were removed.

Lines 146-149 were revised.

**Reviewer 2:** The manuscript document laboratory data as prognostic guide

It would be better to have more information on the clinical course

Including antibiotic treatment, nutritional status and treatment.

Albumin support as needed and skin graft.

**Response to reviewer 2:** ‘’Patients with albumin values below 35 g/L were considered to have hypoalbuminemia. Albumin replacement therapy was administered in the clinical follow-up of patients with albumin values below 25 g/L. Grafting therapy was applied in the follow-up of patients with deep burns if epithelialization was insufficient after debridement’’ was added to the material and methods section.

**Reviewer 3:**

1.	Pediatric patients who died due to burns were excluded from the study because of their low number. This limits the ability to analyze whether CRP-albumin ratio and other markers could predict mortality, which is an important outcome in severe burn cases.

2. The study did not evaluate patients based on burn area localization. This is a significant limitation, as the location of burns can influence healing, complications, and hospitalization duration.

3. Patients with inhalation burns were excluded from the study. These burns often hava a distinct clinical course and outcomes, and excluding them may limit the applicability of findings to all pediatric burn patients.

4. The study focuses on patients treated at a single hospital, which may limit the generalizability of findings to other regions, healthcare systems, or patient populations.

5. The threshold value for the CRP-albumin ratio was derived from the study's ROC analysis. This may lead to overfitting and a need for external validation in larger, independent cohorts to confirm the threshold's applicability.

6. The retrospective nature of the study introduces potential biases, such as incomplete data collection or variability in clinical practices over the study period.

7. The study only analyzed CRP, albumin, and CRP-albumin ratio at admission. Longitudinal changes in these markers could provide more detailed insights into their relationship with the clinical course and outcomes.

8. While the mean age and gender distribution are provided, other demographic or socioeconomic factors that might influence recovery or treatment outcomes are not discussed.

9. The sensitivity (75%) and specificity (61.2%) of the CRP-albumin ratio for predicting hospitalization for more than 20 days are moderate, indicating that the marker alone may not be highly reliable without additional context.

10. Although the sample size calculation ensured adequate power, the exclusion of certain subgroups (e.g., older patients and those with specific comorbidities) may limit the study's scope.

**Response to reviewer 3:**

The first 7th and 10th comments were stated in the limitations section of the article.

Comment 8: A revision was made in the results section in line with the reviewer's suggestion.

Comment 9: Revised in the discussion section.
